# Extracellular Matrix Composition Modulates the Responsiveness of Differentiated and Stem Pancreatic Cancer Cells to Lipophilic Derivate of Gemcitabine

**DOI:** 10.3390/ijms22010029

**Published:** 2020-12-22

**Authors:** Stefania Forciniti, Elisa Dalla Pozza, Maria Raffaella Greco, Tiago Miguel Amaral Carvalho, Barbara Rolando, Giulia Ambrosini, Cristian Andres Carmona-Carmona, Raffaella Pacchiana, Daria Di Molfetta, Massimo Donadelli, Silvia Arpicco, Marta Palmieri, Stephan Joel Reshkin, Ilaria Dando, Rosa Angela Cardone

**Affiliations:** 1 Department of Neurosciences, Biomedicine and Movement Sciences, Biochemistry Section, University of Verona, 37134 Verona, Italy; stefaniafor5@gmail.com (S.F.); elisa.dallapozza@univr.it (E.D.P.); giulia.ambrosini@univr.it (G.A.); cristianandres.carmonacarmona@univr.it (C.A.C.-C.); raffaella.pacchiana@univr.it (R.P.); massimo.donadelli@univr.it (M.D.); marta.palmieri@univr.it (M.P.); 2 Humanitas Clinical and Research Center, IRCCS, Department of Gastroenterology-Laboratory of Molecular Gastroenterology, 20089 Rozzano, Milan, Italy; 3 Department of Biosciences, Biotechnology and Biopharmaceutics, University of Bari, 70126 Bari, Italy; grecorafaella1975@gmail.com (M.R.G.); tiagomac94@gmail.com (T.M.A.C.); daria.dimolfetta@uniba.it (D.D.M.); stephanjoel.reshkin@uniba.it (S.J.R.); rosaangela.cardone@uniba.it (R.A.C.); 4 Department of Biomedical Sciences and Human Oncology, School of Medicine, University of Bari Aldo Moro, 70124 Bari, Italy; 5 Department of Drug Science and Technology, University of Torino, 10124 Torino, Italy; barbara.rolando@unito.it (B.R.); silvia.arpicco@unito.it (S.A.)

**Keywords:** pancreatic ductal adenocarcinoma, cancer stem cells, 3D organotypic cultures, gemcitabine, prodrug, extracellular matrix, chemoresistance

## Abstract

Background: Pancreatic ductal adenocarcinoma (PDAC) is a highly lethal disease. Gemcitabine (GEM) is used as the gold standard drug in PDAC treatment. However, due to its poor efficacy, it remains urgent to identify novel strategies to overcome resistance issues. In this context, an intense stroma reaction and the presence of cancer stem cells (CSCs) have been shown to influence PDAC aggressiveness, metastatic potential, and chemoresistance. Methods: We used three-dimensional (3D) organotypic cultures grown on an extracellular matrix composed of Matrigel or collagen I to test the effect of the new potential therapeutic prodrug 4-(N)-stearoyl-GEM, called C18GEM. We analyzed C18GEM cytotoxic activity, intracellular uptake, apoptosis, necrosis, and autophagy induction in both Panc1 cell line (P) and their derived CSCs. Results: PDAC CSCs show higher sensitivity to C18GEM treatment when cultured in both two-dimensional (2D) and 3D conditions, especially on collagen I, in comparison to GEM. The intracellular uptake mechanisms of C18GEM are mainly due to membrane nucleoside transporters’ expression and fatty acid translocase CD36 in Panc1 P cells and to clathrin-mediated endocytosis and CD36 in Panc1 CSCs. Furthermore, C18GEM induces an increase in cell death compared to GEM in both cell lines grown on 2D and 3D cultures. Finally, C18GEM stimulated protective autophagy in Panc1 P and CSCs cultured on 3D conditions. Conclusion: We propose C18GEM together with autophagy inhibitors as a valid alternative therapeutic approach in PDAC treatment.

## 1. Introduction

Pancreatic ductal adenocarcinoma (PDAC) is the fourth most common cause of cancer-related deaths [1]. The incidence and death rates continue to increase, thus predictively rendering PDAC to become the second most frequent cause of cancer-related death by 2030 [2]. PDAC prognosis is very poor, with an overall 5-years survival rate around 5–7% after diagnosis, which is often made when metastatic events have occurred [3,4]. Only a minority of patients (≈20%) are suitable for surgery, which remains the only potentially curative option. However, survival rates are far from being encouraging. Therefore, chemotherapy, in combination with surgical resection, is an important strategy to extend overall survival and reduce symptoms, especially for advanced tumor stages [5,6]. Numerous efforts have been made to improve treatments in PDAC. Nevertheless, the therapeutic response is still largely ineffective and transient [7]. Therapeutic failure is due to many factors, including extrinsic [8] or intrinsic [9] resistance to conventional chemotherapy approaches. During the last decades, the standard treatment for advanced PDAC has been the gemcitabine (GEM), a deoxycytidine nucleoside analog, the metabolite of which, GEM 3-phosphate, interferes with tumor growth through its incorporation into DNA. Alternatively, GEM diphosphate can interfere with DNA synthesis and tumor growth through the inhibition of ribonucleotide reductase [10]. GEM therapy, however, only confers a marginal survival advantage to patients, showing efficacy in less than 20% [11]. Similar to other anticancer agents, GEM induces reactive oxygen species (ROS) generation [12], cell cycle blocking in the S phase [13], and apoptosis of pancreatic carcinoma cells by reducing Bcl-2 expression levels and, at the same time, activating caspase-3 and -9 [14]. On the other hand, the main disadvantage of GEM is its rapid deamination to its inactive metabolite, 2′,2′- difluorodeoxyuridine, by cytidine deaminase, resulting in a short in vivo half-life. For this reason, it is generally administered at very high doses. Furthermore, a large number of patients are resistant to this therapy mainly due to the characteristic dense tumor stroma of PDAC [15].

To improve the treatment with GEM, many efforts have been employed in the identification of new compounds that facilitate or potentiate its effect. In the pharmaceutical field, the generation of prodrugs is widely used to optimize and improve the physical, chemical, and pharmacological properties of a drug [16,17,18]. The problems related to poor aqueous solubility, chemical instability, low half-life, and fast metabolism are, for the most part, resolved using the prodrug approach. In this context, a series of lipophilic prodrugs have been previously synthesized by us by linking the 4-amino group of GEM with valeroyl, heptanoyl, lauroyl, and stearoyl linear acyl derivatives to increase its stability and bioavailability [19]. Since the activity of GEM prodrugs has been successfully studied in some types of tumors [20,21], they could represent an innovative and satisfactory therapy in PDAC.

Some PDAC features determine its aggressive behavior and resistance to different therapeutic strategies. Notably, PDAC is characterized by the development of extensive fibrosis, termed desmoplasia, with stromal components outnumbering pancreatic cancer cells [22]. The stromal content is composed of cancer-associated fibroblasts (CAFs), endothelial cells, immune cells, and other components, such as collagen, laminin, and cytokines, forming a dense three-dimensional network of extracellular matrix (ECM) [23]. The role of desmoplastic stroma in PDAC progression is dual and complex, and it is modulated by interactions between cancer cells and resident stromal cells. The tumor stroma functions as a barrier to the tumor, limiting growth, dissemination, vasculature, and preventing the transformation of a neoplastic lesion into an invasive tumor [24,25]. Conversely, this stromal barrier impairs the direct delivery of antitumor drugs to pancreatic cancer cells and plays an important role in the intrinsic resistance to GEM by mediating the innate or acquired modification of genes involved in GEM metabolism [26]. Moreover, this stromal matrix favors the selection of a subpopulation of cells with stem cell properties, namely cancer stem cells (CSCs), and their maintenance in a stemness state [27,28]. It has been shown in different cancer types, including PDAC, that CSCs are responsible for resistance to standard therapy, metastatic potential, and disease relapse following resection surgery [29,30,31,32,33]. CSCs are generally more resistant to treatment than the more differentiated tumor cells [34]. As a consequence, the tumor often is apparently removed after therapy. Nonetheless, usually, it grows back since the rare CSC population has survived. This points out the urgent need to discover combined treatments focusing on the bulk tumor of differentiated PDAC cells as well as on the CSC component. Although the stemness features are partially sustained by intrinsic mechanisms, such as DNA methylation or demethylation and gene mutations, they are strongly enhanced by the crosstalk between CSCs and their surrounding stromal environment [35]. Therefore, it is crucial to study CSCs in the context of their stromal niche and to develop in vitro models that recapitulate the in vivo heterogeneity of the primary tumors and their interactions with the surrounding environment.

Here, we used three-dimensional (3D) organotypic cultures of pancreatic cancer cells and their derived CSCs, growing on Matrigel- or collagen I-rich ECM, as a model of the in vivo interactions between tumor cells and their changing surrounding matrix/stroma. Indeed, Matrigel is representative of an early tumor stage, whereas collagen I-rich ECM more likely reflects the stroma of an advanced PDAC [36,37].

With these tumor–stroma models of PDAC, we evaluated how the different ECM compositions influence the cellular response to treatment with GEM and, especially, with its lipophilic prodrugs obtained by conjugating GEM with the fatty acid chains, (4-(*N*)-lauroyl-GEM, C12GEM) and (4-(*N*)-stearoyl-GEM, C18GEM). Furthermore, to better characterize the most effective prodrug, we elucidated the intracellular mechanisms involved in drug uptake that are known for GEM [38] but not completely clear for the lipophilic prodrugs. Defects in apoptotic pathways and deregulation of apoptotic proteins, such as poly-ADP-ribose polymerase 1 (PARP1) and apoptosis-inducing factor (AIF), play decisive roles in the development of PDAC [39]. Therefore, strategies that aim to re-establish the apoptotic process are the basis of the activity of anticancer drugs. Finally, since autophagy may act either as a defense mechanism or as a death mechanism [40,41], we investigated the regulation of cell death and autophagy by GEM prodrugs and propose C18GEM together with an autophagy inhibitor as a valid alternative therapeutic approach in PDAC treatment.

## 2. Results

### 2.1. C18GEM Is More Effective Than GEM in Inhibiting PDAC CSCs’ Growth in Both 2D and 3D Conditions

**Figure 1:**
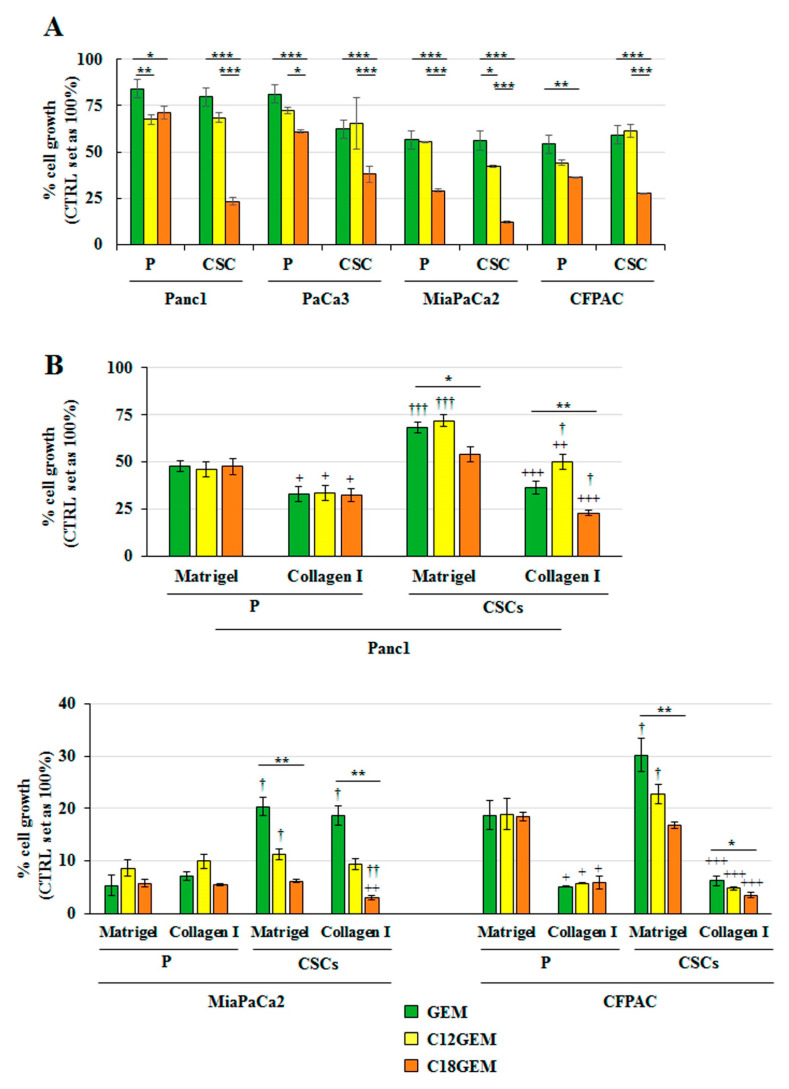
4-(*N*)-stearoyl- Gemcitabine (C18GEM) is more effective than Gemcitabine (GEM) in the inhibition of pancreatic ductal adenocarcinoma cancer stem cells’ (PDAC CSCs) growth. Cell viability analysis of PDAC parental (P) cells and CSCs treated with 50 μM of GEM or C12GEM, or C18GEM for 72 h in two-dimensional (2D) conditions (**A**) or for 7 days in Matrigel- and collagen I-rich extracellular matrix (ECM) (**B**). Cell viability was measured by Resazurin Cell Viability Assay Kit, as described in Materials and Methods. Values are the means (±SE) of at least three independent biological replicates. Statistical legend: *p* < 0.05 (*), *p* < 0.01 (**), or *p* < 0.001 (***), as indicated by bar in figure; *p* < 0.05 (+), *p* < 0.01 (++), or *p* < 0.001 (+++) collagen I versus Matrigel in each cell line for each drug; *p* < 0.05 (†), *p* < 0.01 (††), or *p* < 0.001 (†††) CSCs versus parental (P) cells in the two ECMs for each treatment.

The antitumor activity of GEM and its lipophilic prodrugs, C12GEM and C18GEM, was evaluated on different PDAC cell lines, including Panc1, PaCa3, MiaPaCa2, and CFPAC grown in two-dimensional (2D) and Panc1, MiaPaCa2, and CFPAC grown in organotypic 3D cultures, that we called parental (P) cells, and on the derived CSCs. As shown in Figure 1, cells responded differently to treatments depending on their culture conditions. Indeed, when cells were seeded in 2D (Figure 1A), GEM similarly restrained cell growth in both P cells and CSCs compared to controls (set at 100%). Instead, the lipophilic prodrugs were generally more successful than GEM on both cell types and, especially, C18GEM drastically reduced CSC growth, indicating a higher sensitivity of the CSCs toward C18GEM. When CSCs and P cells were seeded on the 3D organotypic setup consisting of a Matrigel- or a collagen I-rich ECM (Figure 1B), we found that CSCs grown on Matrigel were more resistant than P cells to both GEM and C12GEM. Since Panc1 was the most resistant cell line both in 2D and 3D conditions and C18GEM appeared to be the most promising prodrug in terms of efficacy, especially towards the CSC compartment, which is known to be responsible for chemoresistance and tumor relapse, we focused all subsequent experiments on the comparison between GEM and C18GEM in Panc1 cells.

### 2.2. Intracellular Uptake Mechanisms of GEM and C18GEM in Panc1 P and Panc1 CSCs

**Figure 2:**
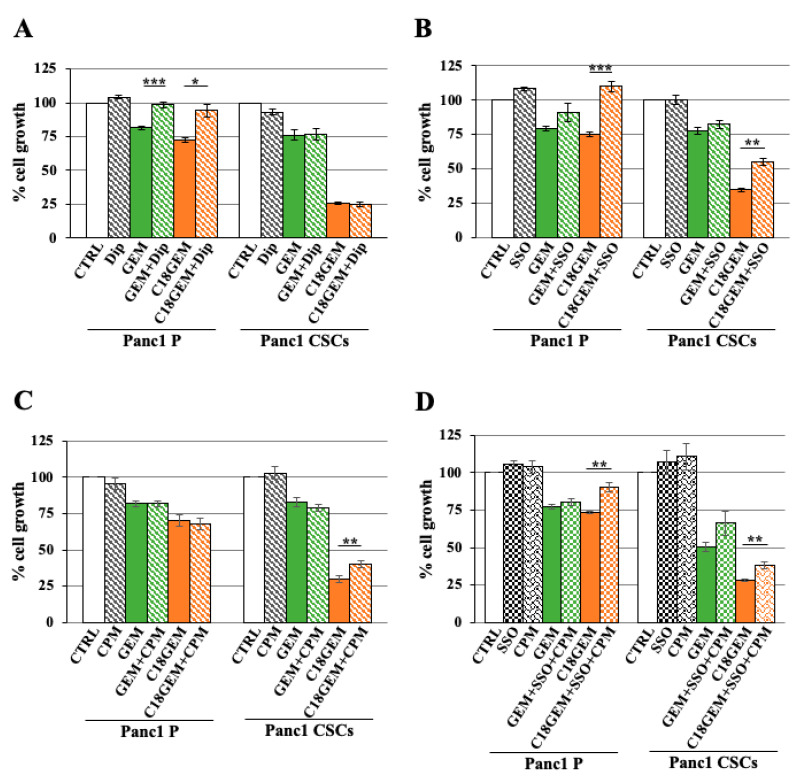
Intracellular uptake mechanisms of GEM and C18GEM in Panc1 parental and Panc1 CSCs. Cell viability analysis of Panc1 parental (P) cells and CSCs treated with 50 μM of GEM or C18GEM for 72 h in 2D conditions in the absence or presence of 10 μM dipyridamole (Dip) (**A**) or of 10 μM sulfo-N-succinimidyl oleate (SSO) (**B**) or of 0.5 μM chlorpromazine (CPM) (**C**) or a combination of SSO and CPM (**D**). Cell viability was measured by a Resazurin Cell Viability Assay Kit, as described in Materials and Methods. Values are the means (±SE) of at least three independent biological replicates. Statistical legend: *p* < 0.05 (*), *p* < 0.01 (**), or *p* < 0.001 (***), as indicated in figure.

A wide range of nucleoside-derived antitumor drugs is described to enter the cells through the membrane nucleoside transporters [38]. In particular, hENT1, a member of the equilibrative nucleoside transporters, is considered to be predominantly involved in GEM incorporation [42]. However, the intracellular uptake mechanism for the lipophilic prodrugs of GEM still remains unclear. Here, we investigated different mechanisms using the following membrane entry inhibitors: dipyridamole (Dip), a non-specific inhibitor of membrane nucleoside transporters; sulfo-N-succinimidyl oleate (SSO), an irreversible inhibitor of the fatty acids translocase CD36; chlorpromazine (CPM), an inhibitor of clathrin-mediated uptake, and methyl-β-cyclodextrin (MβCD), an inhibitor of lipid raft formation by cholesterol depletion. Parental cells and CSCs were treated with 50 μM of GEM or C18GEM plus increasing amounts of the inhibitors, and cell growth was measured after 72 h of treatment. We reported only the most effective concentration for each inhibitor among those tested, and the condition with each inhibitor alone was evaluated to exclude their toxicity. We found that in P cells, but not in CSCs (Figure 2A), GEM and its lipophilic formulation were dependent on nucleoside transporters for entering into the cells, as suggested by the increase in cell viability after combined treatment with drugs and dipyridamole. To clarify this substantial difference between the two cell types, we further analyzed the expression levels of hENT 1 and 2 transporters by real time-PCR. We found no relevant expression differences at the mRNA level of either hENT 1 or 2 between P cells and CSCs (Supplementary Figure S1A). Afterward, we investigated the transport mechanism mediated by CD36, the translocase involved in fatty acid uptake. As shown in Figure 2B, the SSO inhibitor did not influence the effect of GEM on cell growth while it determined a total or a partial rescue only in combination with C18GEM in P cells and CSCs, respectively. Subsequently, we analyzed the role of clathrin-mediated endocytosis in intracellular drug uptake. As reported in Figure 2C, CPM partially recovered cell growth inhibition after the combined treatment with C18GEM only in CSCs and not in P cells, while it did not change cell growth after the combined treatment with GEM. Since both SSO and CPM alone partially rescued CSCs’ growth, we analyzed the effect of a double treatment with SSO/CPM in Panc1 cells and CSCs. Figure 2D shows that SSO/CPM, together with C18GEM, did not improve the rescue effect of the treatment with a single inhibitor. A possible explanation might reside in the ligand-bound interaction of CD36 with a range of associated proteins in the membrane to transmit further signals, including clathrin-binding proteins. However, the mechanism is not completely clear [43]. In contrast to these results, the investigation of lipid raft-mediated endocytosis showed that in both cell types, MβCD did not influence the effect of the drugs on cell growth (Supplementary Figure S1B), suggesting that C18GEM and GEM are not dependent on lipid rafts for entering into the cells.

### 2.3. C18GEM Induces a Higher Regulated Cell Death Than GEM in Both Cell Lines Grown on 2D or 3D Cultures

**Figure 3:**
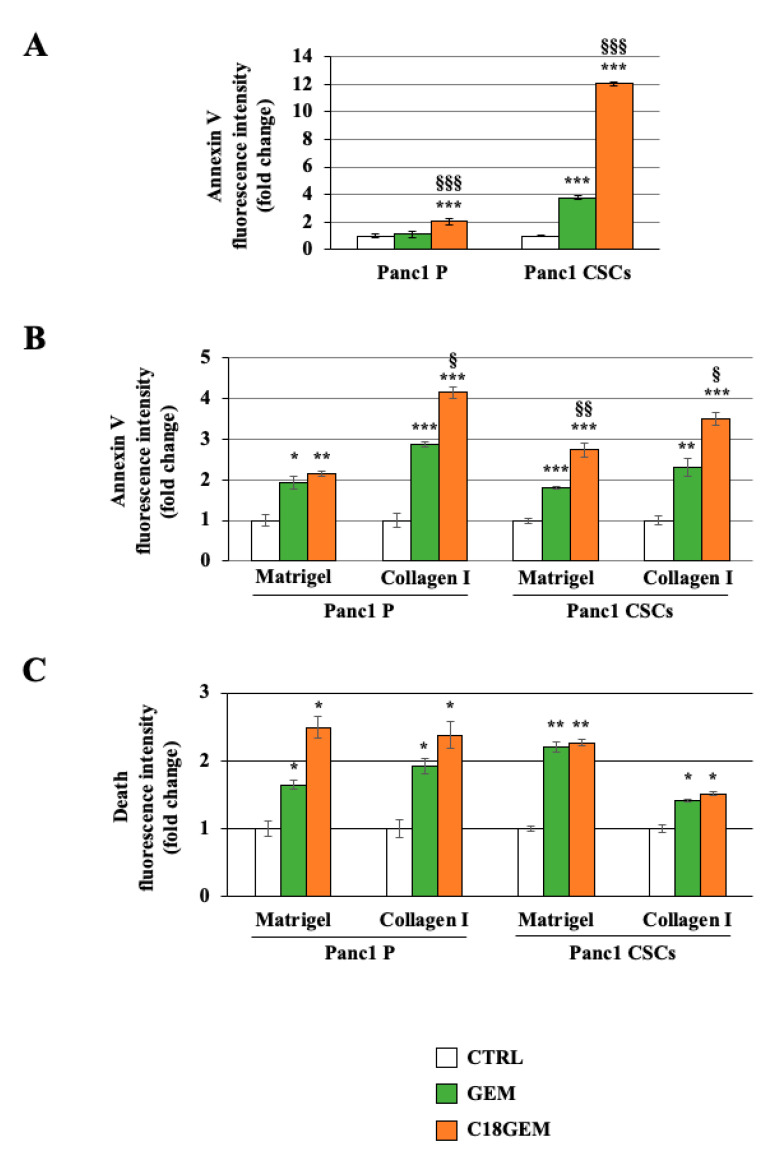
C18GEM induced an increase in regulated cell death compared to GEM in both cell lines grown on 2D or three-dimensional (3D) cultures. Annexin V fluorescence intensity in Panc1 parental (P) cells and CSCs treated with 50 μM of GEM or C18GEM for 48 h in 2D conditions (**A**) or 72 h in Matrigel- and collagen I-rich ECM, as described in Material and Methods (**B**). Ethidium homodimer fluorescence intensity of P cells and CSCs treated with 50 μM of GEM or C18GEM for 72 h in Matrigel- and collagen I-rich ECM (**C**). The values are reported as fold change relative to untreated cells and are the means (±SE) of at least three independent biological replicates. Statistical legend: *p* < 0.05 (*), *p* < 0.01 (**), or *p* < 0.001 (***) GEM or C18GEM versus CTRL; *p* < 0.05 (§), *p* < 0.01 (§§), or *p* < 0.001 (§§§) C18GEM versus GEM.

We next analyzed if the increased sensitivity of CSCs to C18GEM compared to GEM could be attributed to an increased cytotoxic effect. First, we investigated the phosphatidylserine exposure, a common event in some mechanisms of regulated cell death (RCD) [44,45], of cells grown in 2D by performing Annexin V staining. As shown in Figure 3A, C18GEM induced a highly significant increase in Annexin V compared to both untreated- (control) and GEM-treated cells in both P cells and CSCs. In accordance with the highest C18GEM-induced growth inhibition, cell death mediated by C18GEM was much stronger in CSCs than in P cells. Then, we also evaluated phosphatidylserine exposure when the two cell lines were grown on 3D cultures of a Matrigel- or a collagen I-rich ECM (Figure 3B). We found that GEM treatment, and even more C18GEM, induced regulated cell death in P cells and CSCs growing on both the ECM types. Interestingly, both cell lines showed a higher number of Annexin-positive cells after GEM or C18GEM treatment when they grew on collagen I-rich ECM, representative of a more malignant tumor ECM, compared to their growth on Matrigel-rich ECM, representative of an initial tumor ECM. We next determined whether GEM and C18GEM also had an effect on necrotic cell death. For this, cells treated for 72 h were incubated with 16 nM of ethidium homodimer, which enters the cells with damaged plasma membranes, consistent with necrosis. As shown by the analysis of the ethidium homodimer fluorescence intensity reported in Figure 3C, both GEM and C18GEM induced necrosis in both cell lines independently of the ECM composition. Interestingly, C18GEM did not further increase necrosis induced by GEM in CSCs. These data indicate that C18GEM induces an increase in RCD compared to GEM in both cell types without further increasing the necrosis induced by GEM.

### 2.4. Induction of a Different Molecular Mechanism of Regulated Cell Death in Parental Cells and CSCs

**Figure 4:**
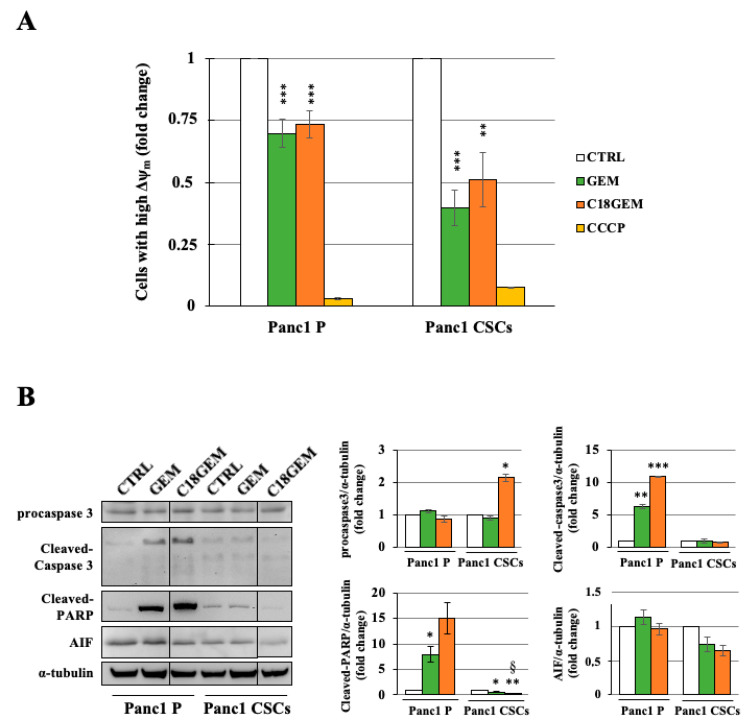
Molecular mechanism of regulated cell death in Panc1 parental cells and Panc1 CSCs. Analysis of mitochondrial membrane potential with JC-1 of Panc1 P cells and CSCs treated with 50 μM of GEM, or C18GEM for 48 h, and the uncoupling compound (CCCP) used as a positive control (**A**). Representative Western blot and quantification of procaspase 3, cleaved caspase 3, cleaved- poly-ADP-ribose polymerase 1 (PARP), and apoptosis-inducing factor (AIF) expression of Panc1 parental (P) cells and CSCs treated with 50 μM of GEM or C18GEM for 48 h in 2D conditions (**B**). The values are reported as fold change relative to untreated cells and are the means (±SE) of at least three independent biological replicates. Statistical legend: *p* < 0.05 (*) or *p* < 0.01 (**) or *p* < 0.001 (***) GEM or C18GEM versus CTRL; *p* < 0.05 (§) C18GEM versus GEM.

**Figure 5:**
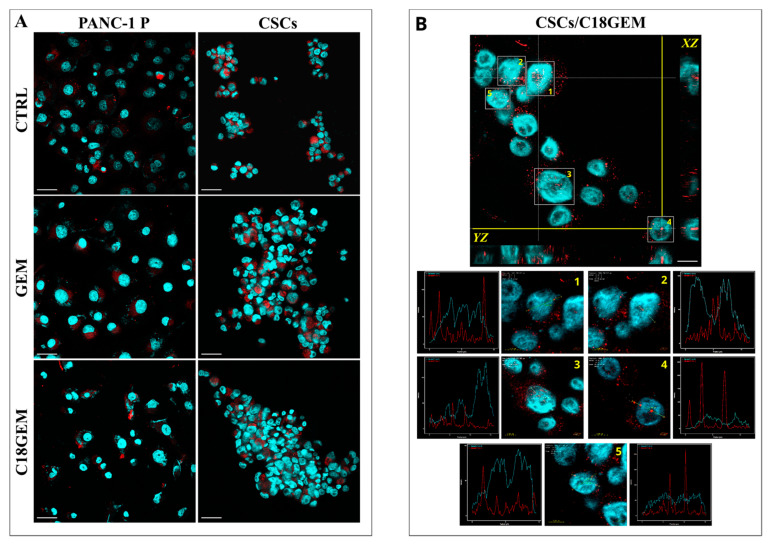
AIF subcellular co-localization. Representative cell images of AIF localization in Panc1 P cells and CSCs treated with 50 μM of GEM or C18GEM for 48 h acquired by a confocal laser-scanning microscope. MIP (maximum intensity projection) images from each analyzed cell line. AIF proteins are red spots and nuclei are in blue. Scale bar, 40 µm (**A**). Representation of CSCs after treatment with C18GEM. In the upper image, the orthogonal sections (ZX and ZY) show AIF protein (red signal) inside the nuclei (blue signal). The nuclei marked with 1, 2, 3, 4, and 5 are shown at higher magnification. For each nucleus, a yellow arrow has been positioned on a red signal. The corresponding plots show the signal intensity (Y axes) and the position identified by the yellow arrow (X-axes). Scale bar, 20 µm and 5 µm (**B**).

To evaluate whether the cell growth inhibition due to C18GEM involved alterations at the mitochondrial level, we investigated their membrane potential. As shown in Figure 4A, C18GEM significantly decreased mitochondrial membrane potential in comparison to untreated cells; however, this effect was similar to that of GEM. To further investigate the molecular mechanisms at the base of the reduced cell growth induced by the drugs, we analyzed by Western blotting the involvement of apoptosis, which is a caspase 3/cleaved-PARP dependent event, and of parthanatos, which is an apoptosis-inducing factor (AIF)-dependent mechanism of cell death. As shown in Figure 4B, the expression of procaspase 3 was not altered after drug treatments, whereas the cleavage of caspase 3 and PARP was increased only in P cells treated with GEM and even more with C18GEM, supporting the induction of caspase-dependent apoptosis in these cells. Then, to investigate the possible involvement of AIF in cell death induced by GEM and its prodrug in CSCs, we analyzed the expression and subcellular localization of AIF. Although the total expression of AIF did not change after drug treatments (Figure 4B), its translocation from the mitochondrion to the nucleus was increased, representing an event linked to parthanatos. In Figure 5, we report representative images of P cells and CSCs treated with 50 μM of GEM or C18GEM for 48 h and incubated with AIF antibody. After drug treatment, we found AIF presence (red spots) in the nuclei only in CSCs, especially in cells treated with C18GEM (Figure 5 and Supplementary Figure S2). Taken together, these data demonstrate that the same drug treatments induce different cell death mechanisms involving caspase 3 in Panc1 P cells and AIF in Panc1 CSCs.

### 2.5. Induction of Autophagy after GEM or C18GEM Treatment in Cells Grown in 3D or 2D Cultures

**Figure 6:**
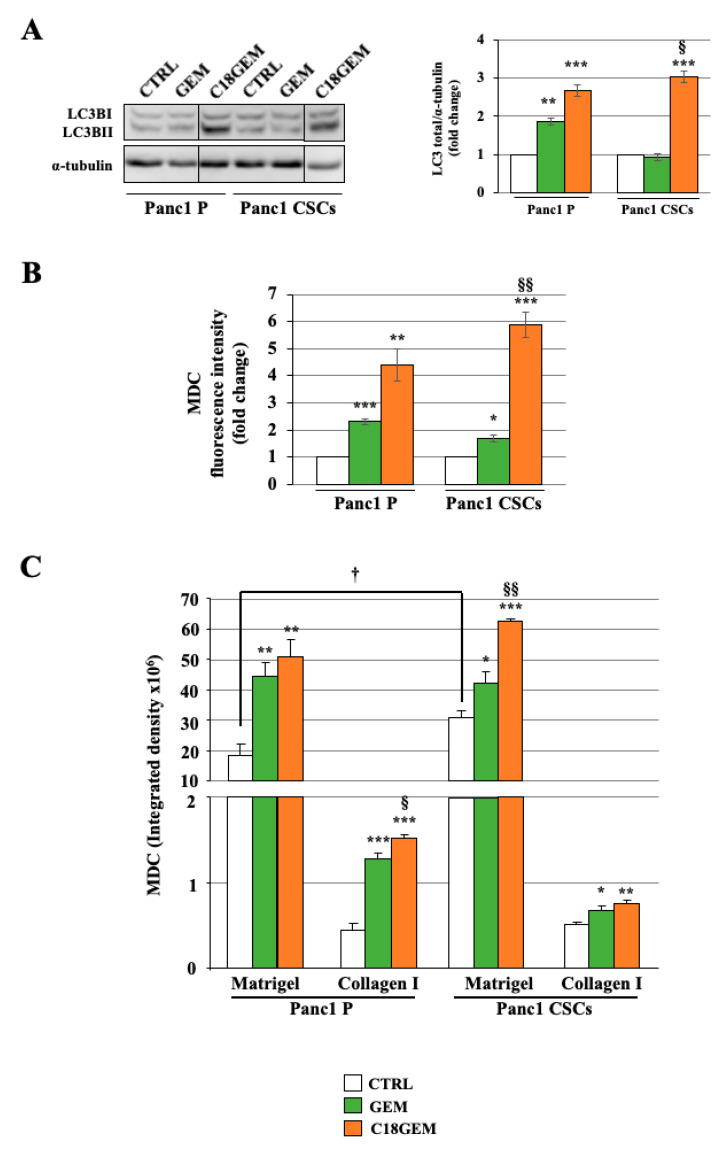
Induction of autophagy after GEM or C18GEM treatment in cells grown in 2D or 3D cultures. Representative Western blot analysis and quantification of LC3-II (**A**). Monodansylcadaverine (MDC) fluorescence intensity accumulated in autophagosomes of parental (P) cells and CSCs treated with 50 μM of GEM or C18GEM for 48 h in 2D conditions (**B**) or 72 h in Matrigel- and collagen I-rich ECM (**C**). The values are reported as fold change relative to untreated cells in 2D conditions and as integrated density in 3D conditions and are the means (±SE) of at least three independent biological replicates. Statistical legend: *p* < 0.05 (*), *p* < 0.01 (**), or *p* < 0.001 (***) GEM or C18GEM versus CTRL; *p* < 0.05 (§), *p* < 0.01 (§§) C18GEM versus GEM; *p* < 0.05 (†) Panc1 P versus Panc1 CSCs.

To better characterize the cell growth inhibition mechanism induced by GEM or C18GEM on P cells and CSCs growing in 2D or 3D cultures, we studied the autophagic response of cells to drug treatments. First of all, we analyzed the expression of LC3-II, the typical autophagic-marker (Figure 6A), which was induced by C18GEM in both cell types. Then, we investigated the number of autophagosomes by labeling the cells with monodansylcadaverine (MDC), a specific marker of autophagic vacuoles. We report the autophagic response to the different treatments of cells growing in 2D cultures (Figure 6B) or on 3D organotypic cultures (Figure 6C). In cells growing in 2D, we found that GEM and especially C18GEM induced MDC uptake, indicating an increased autophagosome formation in both cell lines, with a slightly higher effect in CSCs. When the two cell lines were cultured in 3D, we found that cells growing on the higher Matrigel ECM content had an exceptionally high autophagic ability, and this vacuolated phenotype was even more pronounced in CSCs than in P cells. After drug treatment, the autophagic activity of both cell types increased on both 3D organotypic setup. To confirm autophagy induction by the drugs, we used the autophagy inhibitor chloroquine (CQ) in combination with GEM or C18GEM. The data presented in Supplementary Figure S3A show that autophagy is significantly decreased with CQ in all the treatments. All these findings prompted us to investigate whether the drugs might induce a pro-survival mechanism of autophagy in the cells.

### 2.6. Autophagy Inhibition Sensitizes Parental Cells and CSCs Growing on Different ECM Compositions to Both GEM and C18GEM Treatments

**Figure 7:**
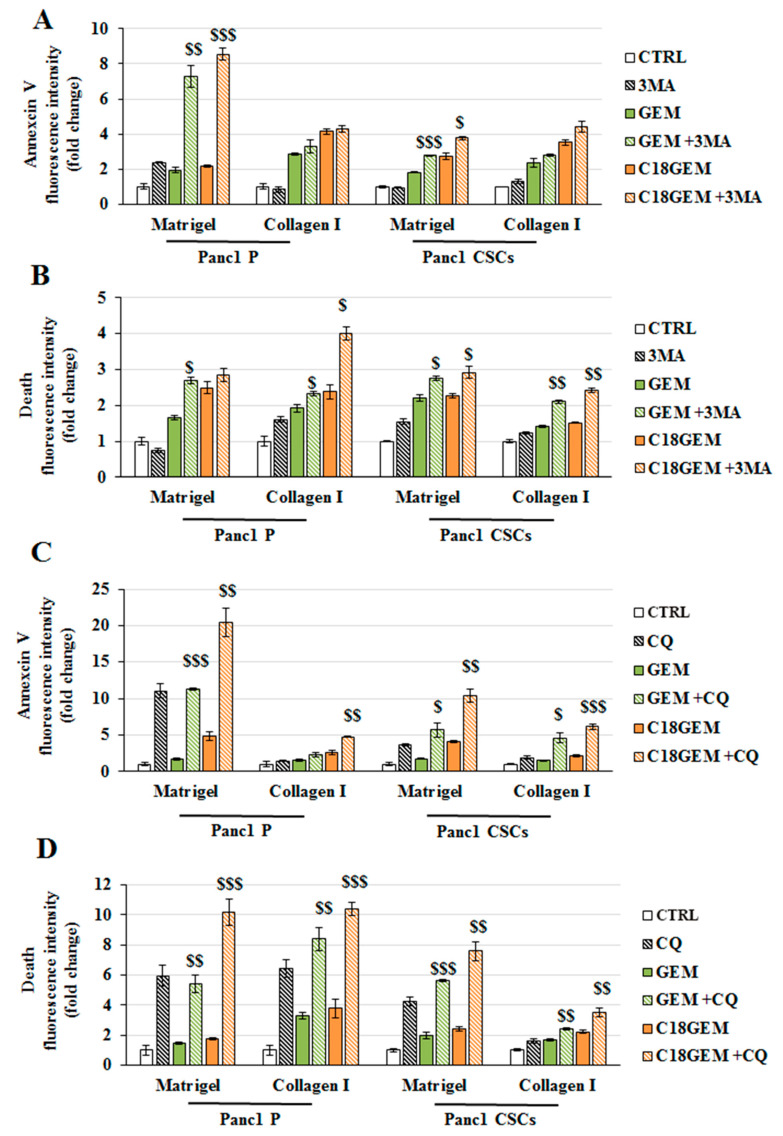
Autophagy inhibition sensitizes Panc1 parental and CSCs growing on different ECM compositions to both GEM and C18GEM treatment. Annexin V (**A**) and ethidium homodimer (**B**) fluorescence intensity of parental (P) cells and CSCs treated with 50 μM of GEM or C18GEM for 72 h in Matrigel- and collagen I-rich ECM in the absence or presence of 3 mM 3-MA, an autophagy inhibitor. Annexin V (**C**) and ethidium homodimer (**D**) fluorescence intensity of parental (P) cells and CSCs treated with 50 μM of GEM or C18GEM for 72 h in Matrigel- and collagen I-rich ECM in the absence or presence of 100 μM CQ. The values are reported as fold change relative to untreated cells and are the means (±SE) of at least three independent biological replicates. Statistical legend: *p* < 0.05 ($), *p* < 0.01 ($$), or *p* < 0.001 ($$$) GEM or C18GEM versus GEM+3-MA or C18GEM+3-MA.

To disclose whether autophagy could act as a pro-survival mechanism used by both cell lines in response to GEM or C18GEM treatments, we tested the autophagy inhibitors 3-methyladenine (3-MA) and chloroquine (CQ) in the absence or presence of the two drugs and measured both Annexin V and ethidium homodimer fluorescence at 3 and 7 days of treatments. As shown in Figure 7A, when cells were cultured on a Matrigel-rich ECM, early cell death induced at 3 days by GEM or C18GEM was strongly increased by the presence of 3-MA, especially in P cells, suggesting that autophagy acts as a pro-survival mechanism on an early tumor ECM. Conversely, when both cell lines were cultured on a collagen I-enriched ECM, the drug-mediated cell death was not affected by autophagy inhibition (Figure 7A). On the other hand, 3-MA increased necrotic cell death induced by drug treatments after 3 days in both cell lines growing on both Matrigel- and collagen I-rich ECM (Figure 7B). A further increase of necrosis was observed at 7 days after the combined treatment with 3-MA and GEM or C18GEM in both cell lines growing on both ECMs (Supplementary Figure S3B). A more evident effect was observed when cells were treated with the drugs in combination with CQ, which is an inhibitor of the last stages of autophagy. Indeed, data presented in Figure 7C,D show that in almost all the conditions, CQ further increased cell death in both cell types. Overall, our data demonstrate that drug-induced regulated cell death and necrosis were further potentiated by autophagy inhibitors 3-MA or CQ, revealing a protective role for autophagy in both cell types growing on organotypic 3D cultures.

## 3. Discussion

A characteristic hallmark of PDAC is a dense fibrotic stroma or desmoplasia, in which two major cell types, CAFs and pancreatic stellate cells, secrete ever larger amounts of collagen I as the tumor progresses [25,36]. This resulting collagen I-rich microenvironment completely embeds and interacts with the tumor cells, supporting malignant progression and resistance to chemotherapy [46,47]. Upregulation of collagen I in the primary tumors and metastatic lesions of PDAC is also directly correlated with poorer outcomes for the patients [48].

The complex PDAC heterogeneity/malignancy, and the presence of a small subpopulation of cells with features of stem cells and self-renewal capacity also participates [49], promotes, and sustains the tumor and its metastatic ability [50]. CSCs have several survival advantages over the differentiated cancer cell population, including high resistance to radio- and chemotherapy [51]. For this reason, research efforts aiming at the development of alternative CSCs-targeted therapeutic approaches are of great importance. The direct targeting of pancreatic CSCs in combination with the killing of the more differentiated cancer cells increases the efficacy of the treatment, as indicated by a longer survival in preclinical xenograft models [52]. Although the stemness features and the acquired resistance of CSCs to treatment are partially sustained by intrinsic genetic and epigenetic alterations, a crucial role in their phenotypic plasticity and their response to therapy is determined by their crosstalk with the surrounding tumor environment [36,53]. Thus, it becomes essential to understand the biology of CSCs and cancer cells in the context of the particular evolving microenvironment in which they reside [36]. Although in vitro 2D platforms are well established and straightforward to use, the obtained results could fail to be translated similarly into in vivo settings [54].

Significant advances over the past decades allow the application of 3D platforms suitable for studying cellular mechanisms and for identifying effective anticancer therapeutics under in vivo-like conditions [55,56]. For this reason, we used 3D organotypic cultures of pancreatic cancer cells and their derived CSCs growing on both a Matrigel-rich ECM or a collagen I-rich ECM that better recapitulate the transition of the native tumor and its surrounding microenvironment from its early development when the tumor cells are mainly exposed to the basal membrane, i.e., Matrigel, to the later stages of tumor progression, when the collagen-I predominates in the tumor ECM.

It is recognized that GEM is rapidly deaminated in blood, liver, kidney, and other tissues, exhibiting a very short half-life [57]. Different approaches have been tried to improve the GEM metabolic stability and its in vivo cytotoxic activity, such as the synthesis of an acyl moiety that protects the drug from rapid inactivation and improves its antitumor activity compared to the pure drug [58]. The GEM 4-(N)-acyl derivatives (C18GEM) activity was studied in vivo on human colorectal adenocarcinoma (HT-29) and nasopharyngeal carcinoma (KB 396p) cells [20], demonstrating higher efficacy than native GEM. Little is still known about the effect of this prodrug approach in pancreatic cancer cells and pancreatic CSCs.

In this work, we demonstrated that the collagen I-rich ECM sensitized both P cells and CSCs to the activity of GEM, C12GEM, and C18GEM. Interestingly, C18GEM was more effective than the other drugs on the Panc1 CSCs, regardless of the matrix, and this growth-inhibitory activity was even stronger when CSCs were cultured on collagen I that mimic the late stage of tumor progression. To explain the greater sensitivity of Panc1 CSCs to C18GEM treatment than the parental cell line, we investigated the mechanism of intracellular transport of the drugs. GEM is described to enter the cells through the membrane nucleoside transporters [38]. However, for the lipophilic prodrugs of GEM, the mechanism of intracellular transport is not completely clear. We found that the two cell types exhibited different intracellular uptake mechanisms of the drugs. In Panc1 parental cell line, in contrast with the derived CSCs, C18GEM and GEM alone are dependent on nucleoside transporters for entering the cells. This is not due to a difference in expression of the hENT 1 and 2 nucleoside transporters at mRNA levels between the two cell lines but to other mechanisms that need to be investigated. In Panc1 CSCs, C18GEM incorporation is in part dependent on fatty acid translocase CD36, which also mediates the transport of C18GEM into P cells, and in part on clathrin-mediated endocytosis. However, this was not the only mechanism underlying the increased sensitivity of CSCs to C18GEM. PDAC cells are notoriously resistant to apoptosis, thereby explaining their aggressive nature and resistance to conventional treatment modalities [59]. Thus, despite P cells and CSCs showing a different mechanism of drug entrance through the cell membrane, when the prodrug tail was eliminated inside the cell, GEM and C18GEM shared the same mechanism of action within the same cell type, but C18GEM exerted a stronger effect than GEM probably due to its higher stability and bioavailability. Indeed, we found that GEM and C18GEM induce regulated death in parental cells and CSCs, both when grown in 2D and 3D conditions, especially on a collagen I-rich ECM. This cell death underlies a different molecular mechanism between P cells and CSCs. Indeed, we found that drug treatments induced caspase-dependent apoptosis in P cells and an AIF-dependent parthanatos in CSCs. Parthanatos is a cell death mechanism, which is induced by a lethal PARP-1 activation and is only dependent on the AIF translocation from mitochondria to the nucleus [45,60,61]. Both regulated cell death mechanisms (parthanatos and apoptosis) induce phosphatidyl serine exposure that is quantified by AnnexinV analysis [45]. However, the different cell death mechanisms induced by C18GEM would find an explanation based on the different metabolic properties of the cells. Indeed, we recently demonstrated that Panc1 CSCs show different metabolic properties in comparison to P cells [62]. Thus, since it has been shown by other scientists that there exists a link between AIF regulation and mitochondrial activity dysfunction [63], it would be conceivable that the mitochondrial setup of CSCs may influence the induction of a different cell death mechanism respective to P cells. However, further demonstrations are needed to confirm this hypothesis.

The relationship between autophagy and chemoresistance is an emerging field in different types of malignancies, including PDAC [64], and there are several studies demonstrating that autophagy might have a pro-tumor effect conferring survival advantages to cancer cells [65,66,67]. Furthermore, in the last decade, it has been observed, using different cancer models, that autophagy can be a crucial factor for CSCs survival and resistance [68,69]. In this study, we demonstrated that inhibition of autophagy using 3-MA or CQ increased regulated cell death by GEM or C18GEM of P cells and CSCs grown on a Matrigel-rich-ECM. Moreover, 3-MA and CQ were also able to increase necrotic cell death after drug treatments in both cell types grown on a Matrigel- and collagen I-rich ECM. Therefore, autophagy might be a cell defense mechanism to escape treatments by blocking cell death mechanisms.

Taken together, our data highlight the possibility of using lipophilic derivatives of GEM, which display higher efficiency in killing CSCs, together with autophagy inhibitors as a therapeutic strategy in the treatment of PDAC.

## 4. Materials and Methods

### 4.1. Cell Lines

Pancreatic adenocarcinoma cell lines, i.e., Panc1, MiaPaCa2, PaCa3, and CFPAC, were grown in RPMI 1640 supplemented with 10% FBS, 2 mM glutamine, and 50 µg/mL gentamicin sulfate (ThermoFisher Scientific, Waltham, MA, USA) and were kept at 37 °C in humidified air containing 5% CO_2_.

CSCs were generated as previously described [70] and cultured in CSC medium, (DMEM/F-12 (US Biological Life Sciences, Salem, MA, USA) supplemented with 1g/l glucose, B27 (ThermoFisher Scientific, Waltham, MA, USA), 1 µg/mL Fungizone (ThermoFisher Scientific, Waltham, MA, USA), 1% penicillin/streptomycin (ThermoFisher Scientific, Waltham, MA, USA), 5 µg/mL heparin (Sigma–Aldrich, St. Louis, MO, USA), 20 ng/mL EGF (epidermal growth factor, Peprotech, Rocky Hill, CT, USA), and 20 ng/mL FGF (fibroblast growth factor, Peprotech, Rocky Hill, CT, USA) at 37 °C with 5% CO_2_.

### 4.2. Prodrug Synthesis and Stability

GEM lipophilic prodrugs were synthesized according to Immordino et al. [19]. The stability of the C18GEM prodrug was assessed in human serum and in complete DMEM/F12 and RPMI 1640 cell culture media used with cell lines. A 1 mg/mL solution of C18GEM in DMSO was added to human serum (sterile-filtered from human male AB plasma, Sigma–Aldrich, St. Louis, MO, USA), to DMEM/F12 or to RPMI 1640 in glass tubes to obtain the 50 μM final concentration. In each condition, the experiments were performed in triplicate. The resulting solutions were incubated at 37 ± 0.5 °C for 24 h. At appropriate time intervals (0, 24, 48, and 72 h), 200 µL of reaction mixtures were withdrawn and added to 200 µL of CH_3_CN containing 0.1% TFA. The samples were vortexed, sonicated for 3 min, and then centrifuged for 5 min at 2500× *g*. The clear supernatant was filtered by 0.45 µm PTFE (Alltech, Nicholasville, KY, USA) and analyzed by RP-HPLC. RP-HPLC procedure allowed the quantification of the unmodified C18GEM prodrug. Calibration curve was obtained with standard solutions of C18GEM (r^2^ > 0.99): seven point calibration standards (1, 5, 10, 20, 25, 50, 100 µg/mL) were prepared by dilution from 1 mg/mL stock solutions (in DMSO) of compound in a mixture of CH_3_CN 0.1% TFA/water 0.1% TFA, 50/50. Analyses were performed with an HP 1200 chromatograph system (Agilent Technologies, Palo Alto, CA, USA) equipped with an injector (Rheodyne, Cotati, CA, USA), a quaternary pump (model G1311A), a membrane degasser (model G1322A), a multiple wavelength UV detector (MWD, model G1365D), and a fluorescence detector (FL, model G1321A) integrated into the HP1200 system. Data analysis was processed using an HP ChemStation system (Agilent Technologies, Santa Clara, CA, USA). The analytical column was an AQUASIL C18 (200 × 4.6 mm, 5 µm; ThermoFisher Scientific, Waltham, MA, USA); the mobile phase consisted of CH_3_CN 0.1% TFA (solvent A) and water 0.1% TFA (solvent B), at a flowrate of 1 mL/min with gradient conditions: 10% A until 4 min, from 10 to 90% A between 4 and 10 min, 90% A between 10 and 20 min, and from 90 to 15% A between 20 and 25 min. The injection volume was 20 µl (Rheodyne, Cotati, CA, USA). The column effluent was monitored at 250 nm referenced against 800 nm wavelength. After 24 h of incubation in human serum, C18GEM prodrug was present at 74% of the unmodified prodrug, according to [19]. In complete DMEM/F12 and RPMI 1640 cell media, the compound displayed good stability during 72 h (86% of the unmodified prodrug in DMEM/F12 and 89% of the unmodified prodrug in RPMI 1640). These results are reported in Supplementary Figure S4.

### 4.3. Organotypic 3D Cultures

Matrigel (Corning Matrigel Growth Factor Reduced Basement Membrane Matrix, Phenol Red-Free) was diluted in RPMI 1640 without gentamicin sulfate and FBS at the final concentration of 7 mg/mL and plated in each well. Bovine collagen I (ThermoFisher Scientific, Waltham, MA, USA) was diluted at the final concentration of 3 mg/mL according to the manufacturer’s directions.

The cell culture plates were then incubated at 37 °C with 5% CO_2_ for 1 h to allow the mixture to create a thin gel on the bottom of the wells and after cells were seeded in each well.

### 4.4. Cell Viability Assay

Cell viability was measured by a Resazurin Cell Viability Assay Kit (Immunological Science), following the instructions of the manufacturer’s protocol. In summary, parental cells and CSCs were plated in 96-well plates on top of the extracellular matrix gel prepared as described above or directly on the top of the well (2D cultures). Viable cells were counted by Trypan Blue dye exclusion, and 7 × 10^3^ cells were seeded in each well.

After 24 h, both cell lines were treated as follows: GEM, C12GEM, and C18GEM for 72 h (2D experiments) or 7 days (3D experiments). To determine their mechanism of intracellular transport in 2D cultures, cells were also treated with different membrane entry inhibitors: dipyridamole (Dip, Sigma–Aldrich, St. Louis, MO, USA), methyl-β-cyclodextrin (MβCD, Sigma–Aldrich, St. Louis, MO, USA), chlorpromazine (CPM, Sigma–Aldrich, St. Louis, MO, USA), sulfo-N-succinimidyl oleate (SSO, Cayman).

After treatments, 15 μL of resazurin were added directly in the medium of each 96-well. After about 3 h of incubation at 37 °C, the fluorescent signal was obtained using a Varian Cary Eclipse Fluorescence Spectrophotometer (Agilent Technologies, Santa Clara, CA, EUA), at 535 nm of excitation wavelength and 590 nm of emission wavelength. The data obtained in each treatment were normalized with the respective control group.

### 4.5. Ethidium Homodimer Assay

Cell death analysis was performed through the cell-impermeant death indicator ethidium homodimer-1 (ThermoFisher Scientific, Waltham, MA, USA). The high-affinity nucleic acid stain is weakly fluorescent until bound to the DNA of dead cells and emits red fluorescence. Panc1 and Panc1 CSCs were plated in 96-well plates and cultured in an organotypic 3D model, as described above. After 24 h, cell lines were treated with 50 μM of GEM or C18GEM in combination with 16 nM of ethidium homodimer, added directly in the cell medium. Cell growth and cell death were monitored in the following days. On days 3rd and 7th, cell images were captured using a Nikon Inverted Microscope Eclipse Ti-S at 4× magnification and analyzed using Image J software.

### 4.6. Immunoblot Analysis

Cells were plated in 60 mm culture plates (5 × 10^5^ cells/plate) and treated as described above with GEM and C18GEM. After 48 h, cells were collected, washed in 1X PBS, and resuspended in RIPA buffer, pH 8.0 (150 mM NaCl, pH 8.0; 50 mM Tris-HCl; 1% Igepal; 0.5% Na-Doc; and 0.1% SDS), 1 mM PMSF, 1 mM Na_3_VO_4_, 1 mM NaF, 2.5 mM EDTA, and 1× protease inhibitor cocktail (Calbiochem; Merck Millipore, Burlington, MA, USA) for 30 min on ice. The lysate was centrifuged at 2300× *g* for 10 min at 4 °C, and the supernatant was used for protein quantification. Protein concentration was measured with the Bradford Protein Assay Reagent (ThermoFisher Scientific, Waltham, MA, USA) using bovine serum albumin as a standard. Thirty micrograms of protein extracts were electrophoresed through a 12% SDS-polyacrylamide gel and electroblotted onto PVDF membranes (Merck Millipore, Burlington, MA, USA). Membranes were then incubated for 1 h at room temperature with blocking solution, i.e., 5% low-fat milk in TBST (100 mM Tris, pH 7.5, 0.9% NaCl, and 0.1% Tween-20), and incubated overnight at 4 °C with the following specific primary antibodies: monoclonal mouse procaspase 3 (3:2000 in blocking solution, sc271759, Santa Cruz Biotechnology, Dallas, TX, USA); polyclonal rabbit cleaved caspase 3 (1:1000 in blocking solution, #9661, Cell Signaling Technology, Danvers, MA, USA); goat AIF (3:2000 in blocking solution, Santa Cruz Biotechnology, Dallas, TX, USA); polyclonal rabbit cleaved-PARP (1:1000 in blocking solution, Cell Signaling Technology, Danvers, MA, USA); polyclonal rabbit LC3B (1:1000 in blocking solution, #2775S, Cell Signaling Technology, Danvers, MA, USA); monoclonal mouse α-tubulin (1:1500 in blocking solution, #CP06, Sigma–Aldrich, St. Louis, MO, USA). A horseradish peroxidase-conjugated secondary antibody was used: anti-rabbit polyclonal IgG (1:2000 in blocking solution, #7074, Cell Signaling Technology); anti-mouse polyclonal IgG (1:10,000 in blocking solution, #074-1806, KPL); anti-goat polyclonal IgG (1:10000 in blocking solution, #705-035-003, Jackson ImmunoResearch, Cambridge, UK). Immunodetection was carried out using chemiluminescent HRP substrates (Merck Millipore) and recorded with Amersham Hyperfilm ECL (GE Healthcare, Italy). To quantify cleaved-PARP expression, bands were scanned as digital peaks, and the areas of the peaks were calculated in arbitrary units using the public domain NIH Image software (http://rsb.info.nih.gov/nihimage/) and normalized on alpha-tubulin signal used as control.

### 4.7. Annexin V and Apoptosis Inducing Factor (AIF) Immunofluorescence

Cells were seeded in a 24-well plate on glass cover-slips at a density of 4 × 10^4^/well. After 24 h, cells were treated with 50μM of GEM or C18GEM for 48 h. Cells were then rinsed in PBS and fixed in 4% (*w*/*v*) paraformaldehyde. After blocking in 5% bovine serum albumin (BSA) and 0.05% of Triton-X-100, cells were incubated with Annexin V (ThermoFisher Scientific, Waltham, MA, USA) 1:40 or apoptosis inducing factor (AIF) antibody (Bethyl Laboratories) 1:100 overnight. Alexa Fluor conjugated antibodies (ThermoFisher Scientific, Waltham, MA, USA) were used as secondary antibodies, and nuclei were stained with DAPI.

Annexin V immunofluorescence. For 2D experiments, cover-slips were mounted over slides in AF1 medium (Dako, Germany). Cell images were captured using a confocal laser-scanning fluorescence microscope Leica SP5 (Leica Microsystem, Manheim, Germany) at 63× magnification. Annexin V fluorescence intensity was analyzed using Image J software. For 3D experiments, apoptotic cells were detected using an apoptosis and necrosis quantification kit (Biotium, Hayward, CA, USA), according to the manufacturer’s protocol. Briefly, after 72 h of treatment with 50 μM of GEM or C18GEM, Panc1 and Panc1 CSCs cells were incubated 30 min in the dark at room temperature, with Annexin binding buffer containing Annexin V and Ethidium. Moreover, to block autophagy, cells were also treated with either 3 mM of 3-Methyladenine (3-MA, Sigma–Aldrich, St. Louis, MO, USA) or 100 μM Chloroquine (CQ, Sigma–Aldrich, St. Louis, MO, USA), in combination or not with GEM or C18GEM. After the incubation period, cell images were acquired using a Nikon Inverted Microscope Eclipse Ti-S at 4× magnification. For both 2D and 3D experiments, fields with similar cell numbers (about 50 cells) have been chosen, and the cell fluorescent intensity was analyzed through the software Image J on the same number of fields for all the tested samples. The measured fluorescent intensity was reported as an average of the different fields and was quantified compared to the corresponding controls (fold change) as in the figures.

AIF immunofluorescence. Images were captured using a confocal laser-scanning fluorescence microscope Leica SP5 (Leica Microsystem, Manheim, Germany) at 40× and 63× magnification. Subsequently, each 3D stack image was deconvolved using Huygens Professional software package (version 19.04, Scientific Volume Imaging B.V.; The Netherlands, http://svi.nl). We used a theoretical Point Spread Function (PSF), and we choose the Classical Maximum Likelihood Estimation (CMLE) algorithm. The deconvolved images were visualized by twin slicer mode of Huygens that provides information related to the fluorescence signal and its position inside the cell to show the co-localization of red (AIF) and blue (nucleus) signal. The deconvolved images were then processed by Imaris software (version 9.1). To demonstrate the presence of AIF proteins inside the nuclei, first, we measured the length of the signal (within 0.3–0.6 µm range), and we converted the red signal as red spheres using a rendering model.

### 4.8. Mitochondrial Membrane Potential Assay

The membrane–permeant JC-1 dye (ThermoFisher Scientific, Waltham, MA, USA) was used as an indicator of mitochondrial membrane potential. Cells (Panc1 and Panc1 CSCs) were plated in 60 mm culture plates (7.5 × 10^5^ cells/plate) and treated with 50 μM of GEM or C18GEM. After 48 h, cells were collected, centrifuged, and resuspended in 0.5 mL of warm medium. For the control tube, the cells were incubated with 50 μM of CCCP (Carbonyl cyanide 3-chlorophenylhydrazone, a potent mitochondrial oxidative phosphorylation uncoupler) at 37 °C, 5% CO_2_ for 5 min. Afterward, the cells were incubated with 2 μM of JC-1 at 37 °C, 5% CO_2_, for 20 min. Then the cells were centrifuged and washed with warm PBS. Finally, the cells were analyzed on a flow cytometer (BD FACSCanto, BD Biosciences, Franklin Lakes, NJ, USA) with 488 nm excitation using emission filters appropriate for Alexa Fluor 488 dye and R-phycoerythrin. Dead cells and debris were excluded based upon forward scatter and side scatter measurements, and the CCCP-treated samples were used to perform standard compensation. The data were calculated as the fold change in the percentage of cells with high membrane potential (red cells) between the control and treated samples and are the average of three biological replicates.

### 4.9. Labeling of Autophagic Vacuoles with MDC

The presence of autophagic vacuoles, as a marker of autophagy, was detected by the fluorescent dye monodansylcadaverine (MDC, Sigma–Aldrich, St. Louis, MO, USA). Cells were seeded in 96-well culture plates on both Matrigel and collagen I for 24 h and also treated with 100 μM Chloroquine (CQ, Sigma–Aldrich, St. Louis, MO, USA), in combination or not with GEM or C18GEM for 7 days. In parallel, 5 × 10^5^ cells were plated in 60 mm 2D culture plates and treated as described above. At the end of the treatments, cells were rinsed in PBS and incubated with a 0.05 mM solution of MDC dye for 15 min, and then washed three times in PBS. For 3D cell cultures, the intracellular MDC fluorescence levels were imaged by a Nikon Eclipse TE 2000S epifluorescence microscope (excitation wavelength 380 nm, emission wavelength 525 nm). The MDC-positive fluorescent spots were quantified by the analysis of the integrated density using Image J software. For 2D cultures, cells were collected and analyzed by flow cytometry (BD FACSCanto, BD Biosciences, Franklin Lakes, NJ, USA). Approximately 10,000 gated events were acquired for each sample and analyzed using FlowJo software (TreeStar, Inc., Ashland, OR, USA). Dead cells and debris were excluded based upon forward scatter and side scatter measurements.

### 4.10. Statistical Analysis

The analysis of variance ANOVA (post hoc Bonferroni) or 2-way ANOVA with Tukey’s multiple comparison tests were performed to compare multiple conditions, and the Student’s *t*-test was used for individual group comparison. Differences were considered significant with *p*-values < 0.05.

## Data Availability

Data is contained within the article or supplementary material.

## Supplementary Materials

Supplementary materials can be found at https://www.mdpi.com/1422-0067/22/1/29/s1. Supplementary Figure S1 (A) Expression levels of hENT 1 and 2 transporters in Panc1 parental (P) cells and CSCs by real-time PCR. Values are reported as fold change relative to Panc1 P cells and are the means (±SE) of at least three independent biological replicates. (B) Cell viability analysis of P cells and CSCs treated with 50 μM of GEM or C18GEM for 72 h in 2D conditions in the absence or presence of increasing concentrations of methyl-β-cyclodextrin (MβCD). Cell viability was measured by a Resazurin Cell Viability Assay Kit, as described in Materials and Methods. Values are the means (±SE) of at least three independent biological replicates; Supplementary Figure S2 (A) Representative cell images of AIF localization in Panc1 CSCs untreated or treated with 50μM of GEM for 48 h acquired by a confocal laser-scanning microscope. CSCs orthogonal sections (ZX and ZY) show the red signal (AIF protein) and the blue signal (nucleus). Scale bar, 20 µm. (B) Rendering obtained by Imaris software of Panc1 CSCs treated with 50 μM of C18GEM for 48 h. AIF proteins are represented as red spheres and the nuclei are in blue. In magnified ROI, red spheres that are crossing through the nuclear membrane (white arrows) can be distinguished from those ones present in the cytosol (magenta arrows). Scale bars, 10 µm and 3 µm. Supplementary Figure S3 (A) MDC fluorescence intensity accumulated in autophagosomes of parental (P) cells and CSCs treated with 50 μM of GEM or C18GEM for 72 h in Matrigel- and collagen I-rich ECM. To better evaluate the difference between GEM or C18GEM treatment in comparison with their combination with CQ, the fluorescence intensity was increased. (B) Ethidium homodimer fluorescence intensity of Panc1 parental (P) cells and CSCs treated with 50 μM of GEM or C18GEM for 7 days in Matrigel- and collagen I-rich ECM in the absence or presence of 3 mM 3-MA, an autophagy inhibitor. The values are reported as fold change relative to untreated cells and are the means (±SE) of at least three independent biological replicates. Statistical legend: *p* < 0.05 (*), *p* < 0.01 (**), or *p* < 0.001 (***) GEM or C18GEM versus CTRL; *p* < 0.05 (§), *p* < 0.01 (§§) C18GEM versus GEM; *p* < 0.001 (†††) Panc1 P versus Panc1 CSCs; *p* < 0.05 ($), *p* < 0.01 ($$), or *p* < 0.001 ($$$) GEM or C18GEM versus GEM+3-MA or C18GEM+3-MA. Supplementary Figure S4 Stability profile of the C18GEM prodrug in human serum at 24 h and in complete cellular media DMEM/F12 and RPMI 1640 for 72 h.
