# Supplementary figures and images for: Extracellular Matrix Composition Modulates the Responsiveness of Differentiated and Stem Pancreatic Cancer Cells to Lipophilic Derivate of Gemcitabine
Source: Int J Mol Sci. 2020 Dec 22;22(1):29. doi: 10.3390/ijms22010029 (PMC7792955; doi:10.3390/ijms22010029)

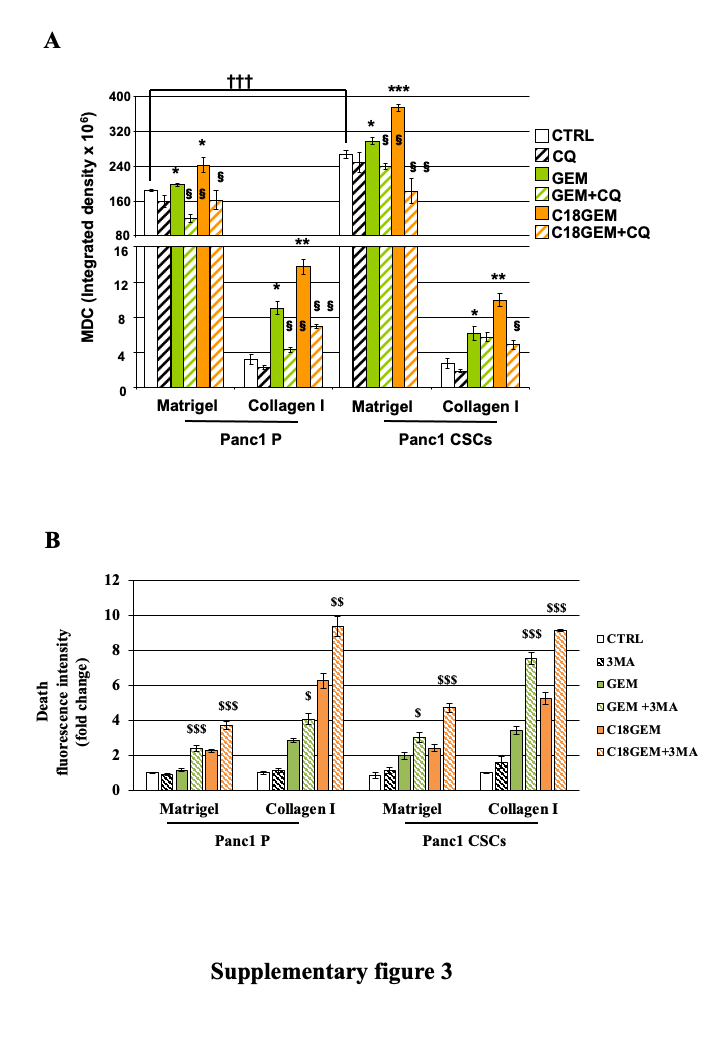

Supplement: Supplementary file 1 [file ijms-22-00029-s001.zip › Suppl. Fig./Suppl fig 3.tiff]

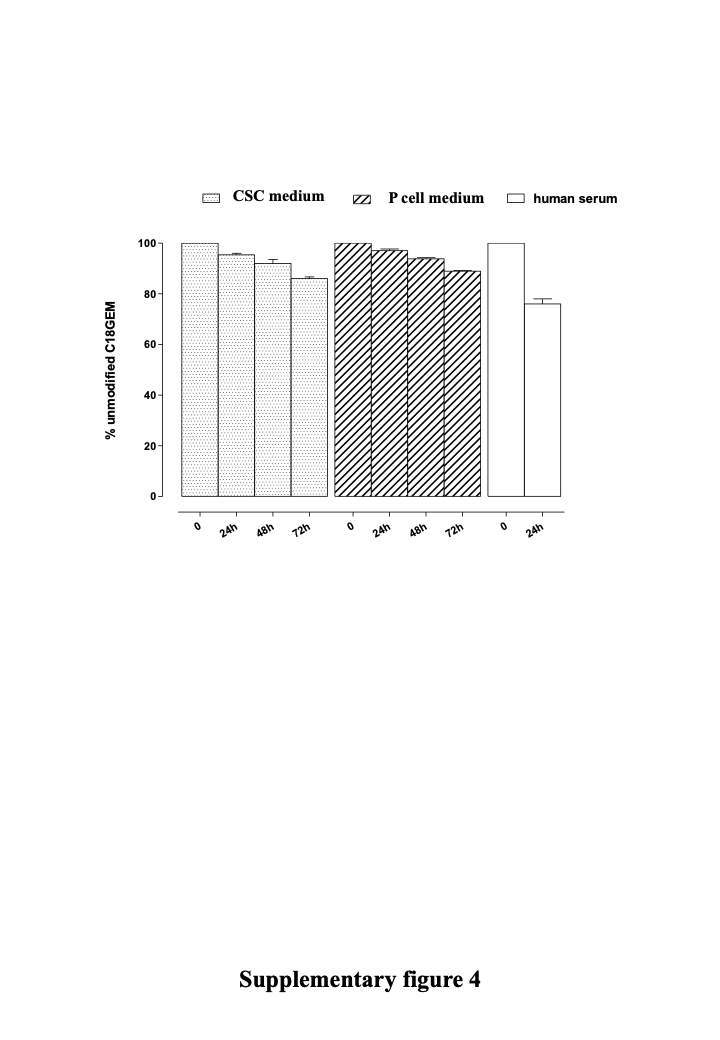

Supplement: Supplementary file 1 [file ijms-22-00029-s001.zip › Suppl. Fig./Suppl fig 4.tiff]

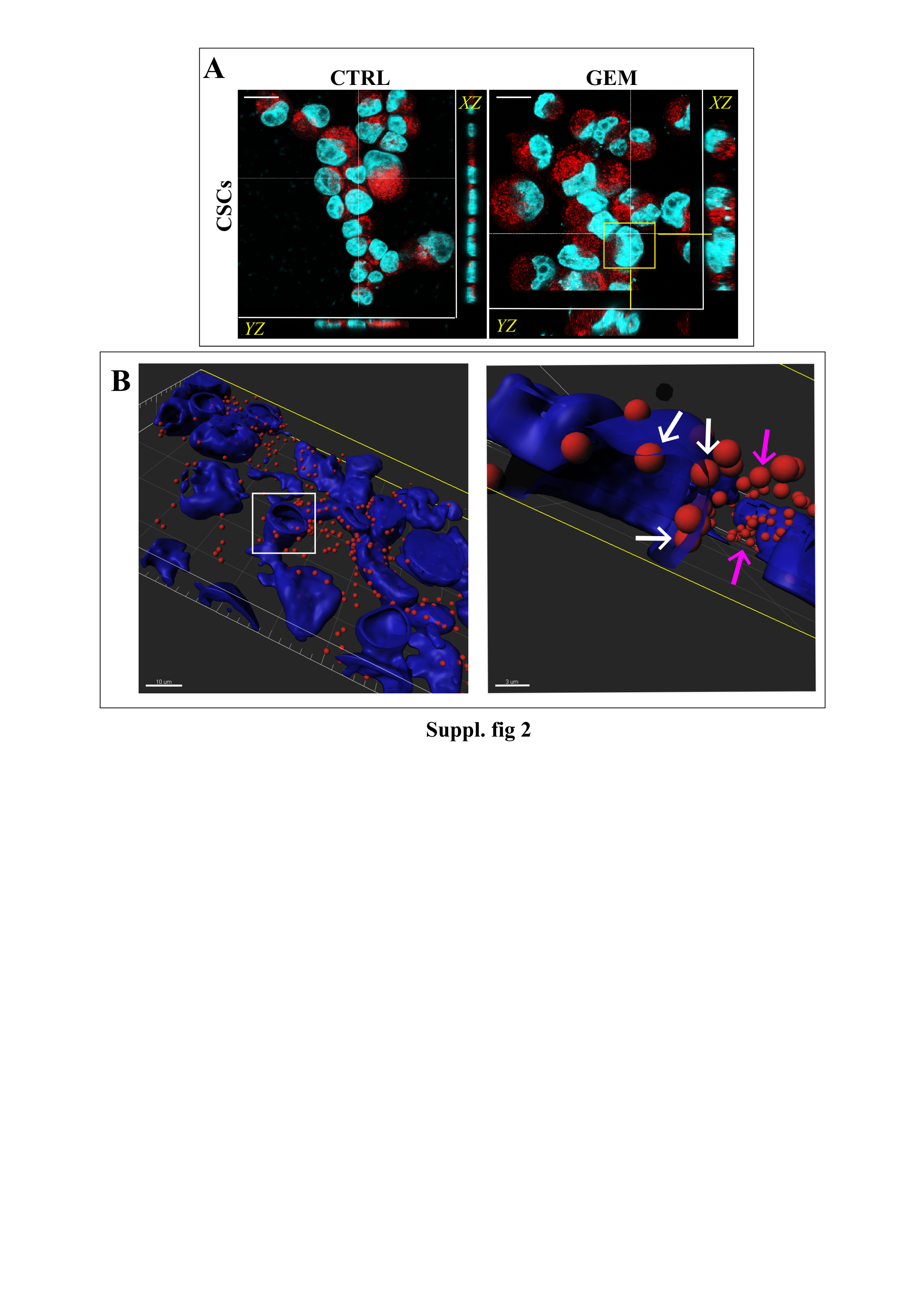

Supplement: Supplementary file 1 [file ijms-22-00029-s001.zip › Suppl. Fig./Suppl fig 2.png]

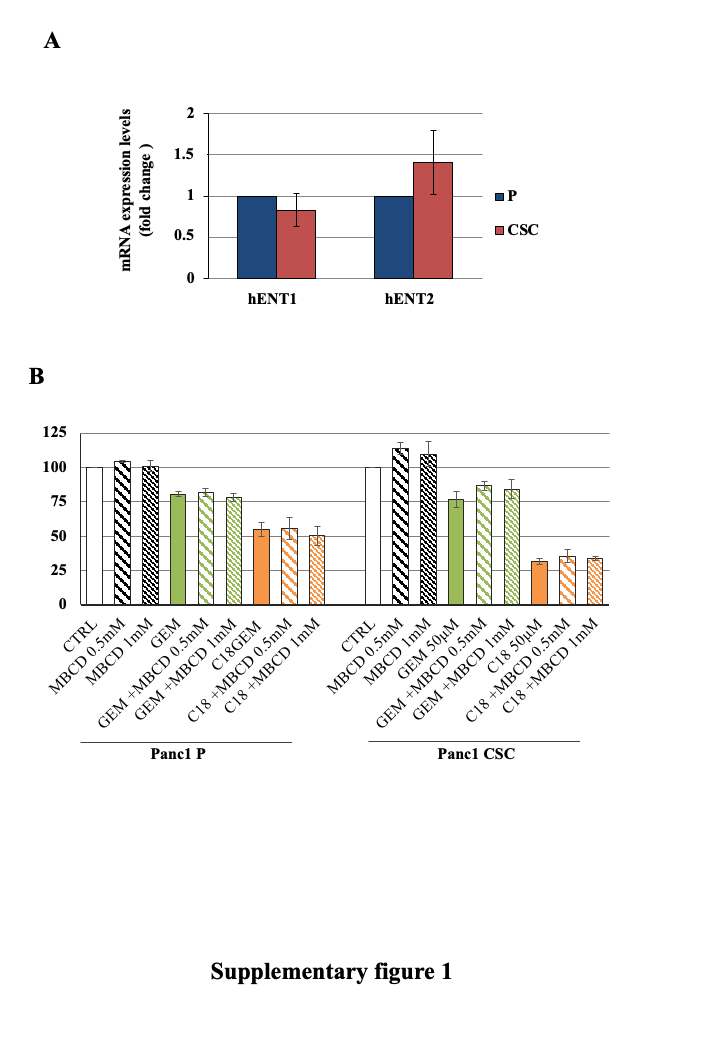

Supplement: Supplementary file 1 [file ijms-22-00029-s001.zip › Suppl. Fig./Suppl fig 1.tiff]
